# Reduction and Maintenance of Scapholunate Dissociation Using the TwinFix Screw

**Published:** 2009-01-29

**Authors:** Razvan C. Opreanu, Michelle Baulch, Abdalmajid Katranji

**Affiliations:** ^a^ Department of Surgery, Michigan State University, East Lansing; ^b^ Michigan Therapy Center, East Lansing; ^c^ Katranji Reconstructive Surgical Institute, East Lansing, MI

## Abstract

**Objective:** Closed reduction and internal fixation of the scapholunate dissociation are currently performed using K-wires or a headless bone screw. We present an alternative for this fixation by means of a cannulated screw with dynamic adjustable interfragmentary compression and discuss the advantages of using this type of technique. **Methods:** Closed reduction of the scapholunate interval was achieved using a K-wire centered through the scaphoid and lunate bones followed by delivery of a cannulated compression screw to maintain the scapholunate interval. **Results:** Nine months after the surgery, the hand active range of motion was significantly improved. Only minimal pain was experienced with high loadings of the wrist and the patient achieved much improvement in his wrist strength. **Conclusions:** The technique we present is simple to perform and represents an attractive alternative to the conventional procedures to achieve the reduction of the scapholunate diastasis and the maintenance of the scapholunate interval.

Scapholunate dissociation is a common injury of the biomechanical linkage between the scaphoid and lunate where the degree of injury may extend from partial or complete disruption of the intrinsic scapholunate interosseous ligament to disruption or attenuation of additional extrinsic stabilizing ligaments.^1^,^2^

Treatment of scapholunate dissociation depends on chronicity of injury, type of instability, etiology, location, direction, pattern, and presence of arthritic changes.^3^ When scapholunate dissociation, scaphoid flexion, or lunate extension with or without capitate descent are present, reduction of the anatomic changes is necessary to be achieved and well maintained to restore the carpal kinematics and allow the recovery of the ligamentous structure and function.

The reduction of the scapholunate interval is commonly performed using multiple K-wires through the scaphoid and lunate bones that could further be used as joysticks. The maintenance of the reduction can be achieved by passing multiple K-wires or a headless bone screw through the scaphoid into the lunate once the bones are in proper anatomic position, with the scapholunate interval and any existent rotational incongruence being reduced.^4^ We report the successful treatment of a case of scapholunate dissociation in which both the reduction and the maintenance of the scapholunate interval were obtained using a cannulated screw with an independent distal rotation system.

## CASE REPORT

The patient is a 68-year-old man who presents to the office following an injury to his right hand. He states that he was picking up a heavy suitcase when he felt a pop and a burning sensation in his right hand. He is right-hand dominant and this injury was severely impacting his activities of daily living.

**Figure 1:**
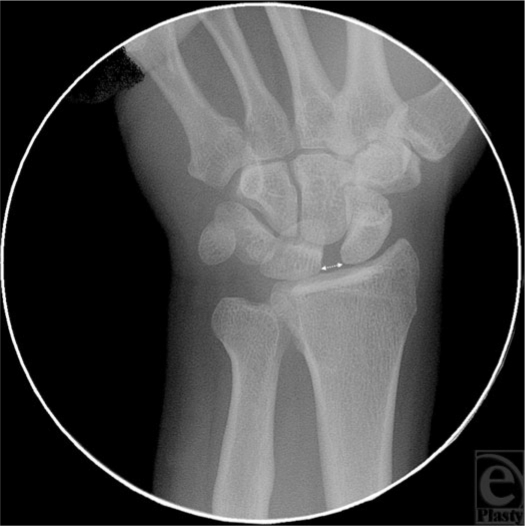
Fluoroscopic image of the carpal rows demonstrating markedly increased scapholunate interval (arrow) with mild palmar flexion of the scaphoid.

Physical examination of the affected wrist revealed maximal tenderness over the dorsum of the wrist and with palpation, tenderness localized to the midcarpal joint. There was moderate swelling to the dorsum of the right wrist and a scaphoid shift test and scapholunate ballottement test were positive. Standard radiographic views of wrist demonstrated an increased scapholunate joint space and minimal rotatory subluxation of the scaphoid (Fig 1). The scapholunate interval measured 4.3 mm, being significantly larger than the interval on the contralateral side, which measured 1.9 mm.

Evaluation of the patient outcome was conducted using subjective and objective parameters. Pain levels were assessed on a numerical rating scale from 1 to 10 (1 being minimal pain and 10 being the worst possible pain). Measurements of the wrist active range of motion and strength were also used for this purpose.

**Figure 2:**
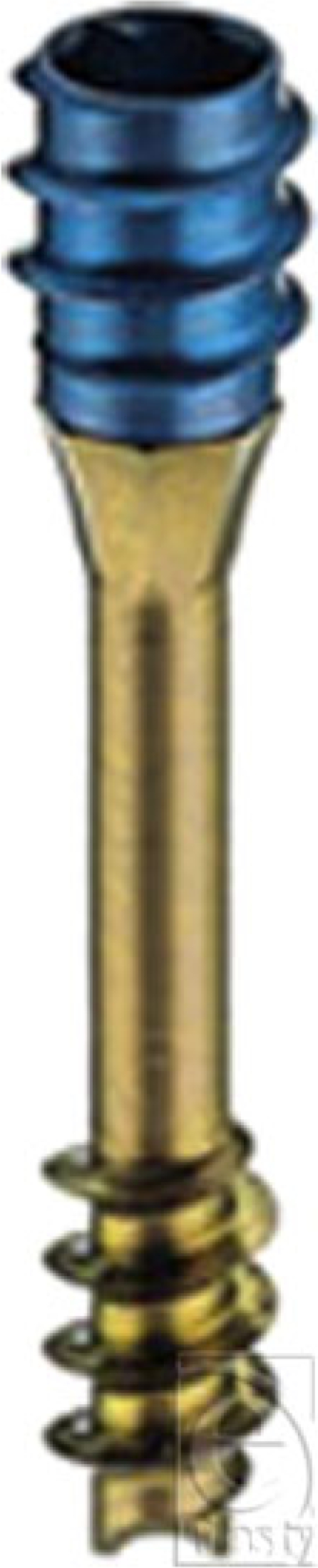
Cannulated double-threaded TwinFix screw.

**Figure 3:**
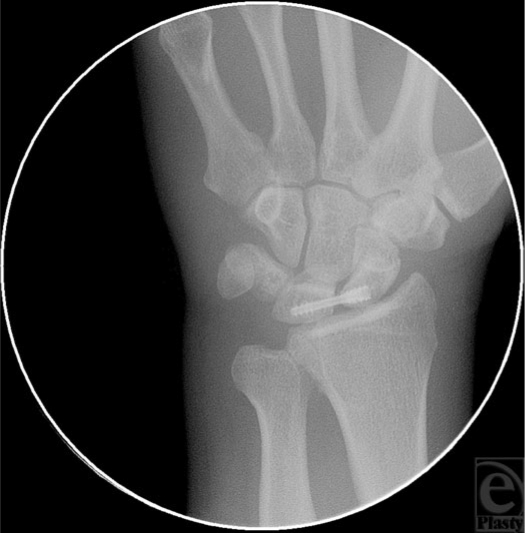
Postoperative fluoroscopic image of the scapholunate interval of the right wrist depicting the reduced scapholunate interval and carpal malrotation.

Conventional diagnostic arthroscopy was performed to assess the degree of ligamentous damage and a scapholunate interosseous ligament tear was identified and subsequently, debridement of the ligament and synovium was conducted. Reduction of the scapholunate diastasis and stabilization of the scapholunate articulation were performed next to allow healing that would further improve or restore the carpal kinematics. A 1-mm K-wire was percutaneously delivered across the scaphoid starting with the distal pole of the bone. Using this K-wire as a joystick, the reduction of the rotatory subluxation of the scaphoid was performed and subsequently, advancement of the K-wire was continued into the lunate. At the point of skin contact of the K-wire, a small stab incision (5 mm) was performed to facilitate the insertion of the screw. Under fluoroscopic visualization, the cannulated screw (Fig 2) (TwinFix, Stryker Leibinger, Kalamazoo, MI) was percutaneously delivered into the scaphoid and lunate, using the K-wire as a guidewire. Once the distal thread of the screw passed into the lunate and the proximal part of the screw was completely in the distal pole of the scaphoid, the proximal part of the screw was locked in place. Using the same screwdriver, isolated rotation only of the distal thread was performed, the distal part of the screw continuing to advance into the lunate, bringing the lunate closer to the scaphoid bone and permitting a dynamic and controlled reduction of the scapholunate diastasis. Rotation of the distal thread of the screw was carried out to the point where a suitable scapholunate interval reduction was attained (Fig 3). A radiograph of the contralateral hand is usually useful for this purpose and a scapholunate interval similar to the left hand was attained. After the proper placement of the screw was obtained, the K-wire was removed.

The procedure was performed as outpatient surgery and a cock-up splint was used for 3 to 4 weeks after the surgery. The patient was permitted to perform activities of daily living but without excessive use of the affected hand. Two weeks postoperatively, occupational therapy was started to maximize early active motion of the wrist.

Postoperative evaluation 9 months after the surgery revealed a decrease in the level of wrist pain from 7 to 1. The changes in the wrist active range of motion and strength with regard to the pre- and postoperative period are depicted in Table 1.

## DISCUSSION

The TwinFix screw is mostly used for fixation of the scaphoid fractures where the main advantage derives from the high compression forces achieved across the scaphoid fragments.^5^ Similarly, considering the lunate and the scaphoid as the bony fragments, the advantages of a dynamic intra-articular compression screw were extended toward the reduction and maintenance of scapholunate diastasis in this case.

Nine months after the surgery, the wrist measurements for active range of motion were improved and the patient regained significant strength in his right wrist. Overall the patient experienced only minimal pain with significant loading of the wrist and the outcome of the treatment was considered good to excellent.

A common feature of the headless bone screw and the TwinFix screw is cannulation to allow optimal reduction and secure screw fixation. The major advantage for the TwinFix screw is the double-independent compression system with the possibility to turn the distal part of the screw separately from the proximal one. This translates into an advantage by applying additional compression forces to the bony fragments,^5^ which offered to our case a well-controlled reduction and maintenance of the scapholunate diastasis. A potential complication associated with the use of this type of screw would be the failure of the hardware that could emerge with significantly high loadings of the wrist. However, this complication is associated with the use of any K-wire or screw and is not specific to this technique.

The procedure carried out in this case is user-friendly and easy to perform. It proves to be a useful alternative to the conventional techniques. The use of a single K-wire theoretically allows a better preservation of the integrity of the scaphoid and lunate bone, whose vascularization could be eventually compromised with the use of multiple K-wires. However, the main advantage of this technique over other common procedures comes from the double-independent compression system that allows for a controlled reduction of the scapholunate diastasis.

## Figures and Tables

**Table 1:** Preoperative and postoperative wrist active range of motion and strength*

Measurement	Left hand	Right hand preoperative	Right hand postoperative (9 mo)
Wrist flexion	70	17	53
Wrist extension	71	15	59
Grip	96	15	72
Lateral pinch	30	8	25
3-point (jaw) pinch	19	1	19
2-point (tip) pinch	13	2	11

*Flexion and extension are measured in degrees of motion. Wrist strength measurements are expressed in pounds.
